# Evaluation of the international forum on evidence informed health policymaking: Addis Ababa, Ethiopia – 27 to 31 August 2012

**DOI:** 10.1186/1478-4505-12-14

**Published:** 2014-03-19

**Authors:** Justin Neves, John N Lavis, Ulysses Panisset, Markus Hultstrand Klint

**Affiliations:** 1 McMaster University, Michael G. DeGroote School of Medicine, 1280 Main St. West, MDCL-3107, Hamilton, ON L8S 4K1, Canada; 2 McMaster University, PPD/CHEPA, 1280 Main St. West, CRL-209, Hamilton, ON L8S 4K1, Canada; 3 World Health Organization, 20 avenue Appia, Room E-173, Geneva CH-1211, Switzerland; 4 Department of Public Health and Community Medicine, Gothenburg University, Box 414 405 30, Gothenburg, Sweden

**Keywords:** Conferences, Evaluations, Meetings, Questionnaires

## Abstract

**Background:**

Meetings and conferences are often used as a tool to disseminate information, network with colleagues, and/or set direction for a field of study, but there is little evidence to support whether such events achieve their objectives. This study evaluates the International Forum on Evidence Informed Health Policymaking (EIHP), a three-day meeting held in Addis Ababa, Ethiopia, in 2012, to determine the success of the meeting based on pre-determined objectives.

**Methods:**

The evaluation strategy was developed based on a previously published conference evaluation framework and operationalized as an end-of-conference participant survey that incorporated both process (programme/organization) and outcome measures (potential changes in behaviour).

**Results:**

Sixty seven of approximately 121 attendees filled out a questionnaire (a 55% response rate) and, overall, participants rated the programme components and plenary sessions very highly. The top three benefits reported by participants were: i) sharing experiences and lessons learned (75%); ii) new opportunities for future collaboration (69%); and iii) new knowledge (67%). Conversely, only 25% or less of meeting participants reported an intent to utilize any of the potential benefits highlighted in the questionnaire, with the notable exception of pursuing new opportunities for future collaboration.

**Conclusions:**

The evaluation findings suggest that the International Forum achieved its objectives of sharing experiences with EIHP and providing opportunities for networking among EIHP initiatives, although there are limited prospects for direct improvements to efforts to support EIHP.

## Report

### Introduction

Meetings and conferences are often used as a tool to disseminate information, network with colleagues, and/or set direction for a field of study; however, there is little evidence to support whether these meetings are effective in relation to their objectives [1,2]. While previous studies have examined the effectiveness of small educational meetings (or workshops) [3], international meetings that bring in a diverse group of stakeholders and have similarly diverse objectives have not been evaluated to the same extent. One way to improve our understanding of the impact of these meetings is to increase the value of end-of-conference evaluations through: i) building the capacity of meeting organizers to develop their own evaluation strategies and ii) increasing the dissemination of meeting evaluations through publication in peer-reviewed journals and other avenues beyond just meeting participants.

**Figure 1:**
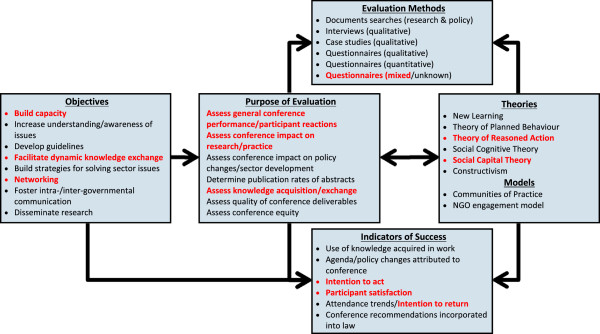
**Conference evaluation framework (adapted from [**[4]**]).** The bulleted components represent the most frequent examples utilized for each category (as reported in the literature). Components highlighted in red indicate which objectives, purpose, methods, and indicators were utilized in the International Forum's evaluation.

In 2012, we published a scoping review to map out what types of objectives and evaluative practices were being utilized at large multi-day meetings [4]. The review culminated in the development of a conference evaluation framework, adapted in Figure 1, to aid the organizers of such meetings in evaluating the impact of their conference on participants and their associated sectors. We utilized this conference evaluation framework to support the development of the evaluation strategy for the International Forum on Evidence Informed Health Policymaking (hereafter referred to as the International Forum).

The International Forum was held in Addis Ababa, Ethiopia, from 27–31 August 2012. The meeting was hosted by Supporting the Use of Research Evidence in African Health Systems (SURE), the WHO Evidence-Informed Policy Network (EVIPNet), and the Regional East African Community Health (REACH) Policy Initiative. The local host was the Ethiopian Health and Nutrition Research Institute (ENHRI). The objectives of the International Forum were: i) to share experiences with (and resources for) evidence-informed health policymaking (EIHP) in low- and middle-income countries (LMICs); ii) to identify opportunities for improving country-level efforts to support EIHP in LMICs; and iii) to provide an opportunity for networking among initiatives to support EIHP [5]. This study aims to examine the strengths and weaknesses of the meeting as a whole (process measures) as well as the potential benefits reported and intent to utilize these benefits (outcome measures) based on the formal feedback from attendees.

## Methods

The International Forum evaluation strategy was operationalized as an end-of-conference participant survey. The survey was adapted from another international meeting with similar attendee demographics and agenda structure. The development of the survey was guided by our previously published conference evaluation framework [4], as well as by consultations with various meeting organizers.

### Data collection

We administered 100 hard copies of the evaluation questionnaire in English (Additional file 1) and 50 copies in French. We placed the questionnaires on participants’ tables prior to the last session of the final day and distributed them at the back of the same conference hall. The questionnaires were collected by volunteers after the session or placed into drop boxes at the back of the hall.

The survey consisted primarily of Likert scale questions evaluating participant satisfaction with the content of the meeting, supplemented by boxes for comments on other strengths or weaknesses of the meeting (process measures). The survey also included checkboxes to collect data on the potential benefits participants received from the meeting and space to report intent to utilize any of these benefits (outcome measures). Finally, we collected information on participants’ previous activities with EIHP (e.g., collaboration between researchers and policy makers when making decisions), both as an indicator of attendees experience with EIHP and to be used as baseline data when following-up with attendees at future iterations of the International Forum to examine any progress in the field as these types of meetings continue.

### Data analysis and synthesis

Two researchers entered the survey data into Microsoft Excel, with double entry of 30% of the data to check inter-rater reliability and reconcile any errors (none were identified). Given the few French responses received, answers written in French were translated into English upon entry and analyzed regardless of initial language. From the data, we calculated the mean and standard deviation of each Likert-scale rating for all survey participants and for the following demographic categories: i) first time at an international meeting on EIHP; ii) from a LMIC; iii) self-reported role; iv) years of experience in EIHP; and iv) gender. These statistics were organized into tables according to overall focus of questions and findings were examined within each demographic category as well as across categories. Qualitative data from comment boxes were analyzed thematically using a constant comparative method. Thematic codes were tallied for each question to highlight which perspectives were most often reported in the survey. Response rates were determined by comparing the self-reported role of participants in the evaluation to the role they reported when registering for the meeting.

We also conducted four multinomial logistic regressions to examine relationships between certain demographic categories of attendees and their intent to utilize the benefits they reported. Regressions were performed in SPSS, with each of the top four benefits reported as dependent variables and the five demographic categories as factors (independent variables). All demographic categories were coded as binary variables except years of experience and self-reported role, which were recoded with dummy variables before being entered into the regression.

## Results

**Figure 2:**
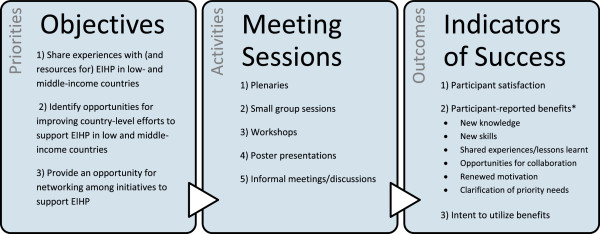
**Condensed logic model for the International Forum evaluation.** *List of all benefits reported can be found in Table 4.

The International Forum was structured as a large consultative process, including hands-on workshops, knowledge sharing sessions, and facilitated discussion, supplemented by five plenaries (Figure 2). The sessions were organized around five themes: i) Evidence informed health policy in action, in particular around sharing experiences related to the process of preparing evidence briefs for policy; ii) Skills training for knowledge translation efforts; iii) Tools to assist in pursuing knowledge translation efforts; iv) People and forming networks to work with highly complex issues and broadly based stakeholders; and v) Collaboration and innovation to improve EIHP initiatives. More detailed names and topics of the meeting sessions can be found in the International Forum’s programme [5].

Of the approximately 121 attendees, 67 filled out the survey (55%), of which 40/75 were researchers (53%), 11/23 policymakers (48%), 4/8 journalists (50%), and 12/15 (80%) attendees who did not report a role. In terms of experience with EIHP (Table 1), journalists had the most experience (by percentage) with the EIHP activities we measured but they were also the smallest grouping of stakeholders at the meeting. Participants, regardless of role, were more likely to undertake activities they could accomplish by themselves (i.e., utilizing research in their policy decision-making) versus approaching other stakeholders (i.e., talking to a researcher/journalist about the available evidence on a policy).

**Table 1:** Number and percentage of participants reporting past experience with evidence-informed health policymaking

**Type of participant**	**Experience (in past 3 months) with evidence-informed health policymaking**	**Number of times action was undertaken**									
		**0**		**1 to 4**		**5 to 9**		**10 +**		**N/A**	
Policymakers (out of 14)	Participants used evidence to support health policy decisions	3	(21%)	5	(36%)	1	(7%)	5	(36%)	0	(0%)
	Participants spoke with researchers about the research evidence available to support policy decisions	2	(14%)	5	(36%)	1	(7%)	2	(14%)	4	(29%)
	Participants spoke with journalists about the research evidence available to support policy decisions	6	(43%)	3	(21%)	2	(14%)	0	(0%)	3	(21%)
Researchers (out of 48)	Participants undertook activities to support health policy decisions	6	(13%)	28	(58%)	8	(17%)	6	(13%)	0	(0%)
	Participants spoke with policymakers about research evidence available to support health policy decisions	10	(21%)	25	(52%)	8	(17%)	3	(6%)	2	(4%)
	Participants spoke with journalists about research evidence available to support reporting on health issues	25	(52%)	16	(33%)	1	(2%)	2	(4%)	4	(8%)
Journalists (out of 7)	Participants searched for evidence to support reporting on health issues	0	(0%)	2	(29%)	3	(43%)	2	(29%)	0	(0%)
	Participants spoke with policymakers about research evidence available to support health policy decisions	1	(14%)	3	(43%)	2	(29%)	0	(0%)	1	(14%)
	Participants spoke with researchers about the research evidence available to support reporting on health issues	0	(0%)	1	(14%)	3	(43%)	2	(29%)	1	(14%)

Overall, the meeting was very well received, with 100% of participants reporting that they would attend the International Forum again in the future. The following sections analyse the specific components of the meeting based on the survey responses. Logistic regressions comparing the different types of sessions and reported benefits (including intent to utilize them) identified no significant correlations.

### Programme

On a scale of 1–5 (very poor to very good), participants rated the overall programme (4.4 [0.7]) and the various types of sessions very highly, which was a consistent finding across all reported demographic categories (Table 2). Participants rated the poster presentations much lower than all other sessions (3.2 [0.9]). Specifically, less experienced attendees as well as journalists and researchers (compared to policymakers) gave lower ratings for the poster presentations.

**Table 2:** Average participant rating with [standard deviation] of programme components

**PROGRAMME (Scale 1–5)**	**Total**		**First time at international meeting on evidence-informed policymaking**		**From low- or middle-income country**		**Self-reported role**						**Years of experience with EIHP**						**Gender (Female)**	
							**Policymaker**		**Journalist**		**Researcher**		**<1**		**1–9**		**10–19**			
	**Mean**	**SD**	**Mean**	**SD**	**Mean**	**SD**	**Mean**	**SD**	**Mean**	**SD**	**Mean**	**SD**	**Mean**	**SD**	**Mean**	**SD**	**Mean**	**SD**	**Mean**	**SD**
Plenary sessions	4.3	[0.7]	4.2	[0.7]	4.2	[0.8]	4.3	[0.6]	4.0	[0.8]	4.3	[0.7]	4.0	[0.7]	4.3	[0.7]	4.6	[0.5]	4.4	[0.7]
Small group sessions	4.5	[0.6]	4.4	[0.7]	4.4	[0.7]	4.1	[0.8]	5.0	[0.0]	4.5	[0.6]	4.7	[0.5]	4.5	[0.6]	4.5	[0.5]	4.5	[0.7]
Poster presentations	3.2	[0.9]	3.0	[1.0]	3.2	[1.0]	3.8	[0.8]	3.0	[1.7]	3.1	[0.9]	2.8	[1.0]	3.3	[0.9]	4.0	[1.0]	3.6	[1.0]
Pre-forum workshop	4.5	[0.7]	4.6	[0.6]	4.5	[0.7]	4.3	[1.0]	5.0	[0.0]	4.5	[0.6]	4.7	[0.5]	4.5	[0.7]	4.8	[0.3]	4.5	[0.7]
Opening and closing dinners	4.2	[0.8]	4.3	[0.8]	4.2	[0.8]	4.3	[0.9]	4.0	[1.4]	4.3	[0.7]	4.0	[0.9]	4.1	[0.7]	4.6	[0.9]	4.1	[0.8]
Possibilities for discussion	4.4	[0.9]	4.3	[0.9]	4.4	[0.9]	4.1	[1.1]	4.4	[0.5]	4.5	[0.8]	4.5	[0.5]	4.4	[0.9]	4.3	[0.5]	4.4	[0.7]
Overall (Programme)	4.4	[0.7]	4.4	[0.7]	4.5	[0.7]	4.5	[0.8]	4.5	[0.6]	4.5	[0.6]	4.6	[0.5]	4.4	[0.7]	4.3	[0.5]	4.5	[0.7]

### Plenaries

Participants rated all five plenary sessions above 4.0 on a 1–5 scale, with the plenary on success stories and lessons learned given the highest rating (4.4 [0.7]) (Table 3). In terms of specific plenaries, LMIC participants rated the panel discussion a 3.2 [1.0] on average, the lowest score for that session across all categories and the lowest rating by LMIC participants for all plenaries. This corresponds to the feelings of certain participants noted in written comments that there were not enough LMIC speakers. In contrast, researchers were generally more positive in their ratings than policymakers or journalists. A few participants noted in their comments that including more current (“true”) policymakers would improve the plenary sessions.

**Table 3:** Average participant rating (with standard deviation) of plenary sessions

**PLENARIES (Scale 1–5)**	**Total**		**First time at international meeting on evidence-informed health policymaking**		**From low- or middle-income country**		**Self-reported role**						**Years of experience with EIHP**						**Gender (Female)**	
							**Policymaker**		**Journalist**		**Researcher**		**<1**		**1–9**		**10–19**			
	**Mean**	**SD**	**Mean**	**SD**	**Mean**	**SD**	**Mean**	**SD**	**Mean**	**SD**	**Mean**	**SD**	**Mean**	**SD**	**Mean**	**SD**	**Mean**	**SD**	**Mean**	**SD**
Opening plenary and welcome	4.1	[0.7]	4.1	[0.7]	4.2	[0.8]	3.9	[0.7]	3.5	[0.6]	4.2	[0.6]	4.1	[0.6]	4.2	[0.6]	3.9	[0.7]	4.3	[0.6]
Looking at EIHP initiatives	4.3	[0.7]	4.2	[0.8]	4.4	[0.7]	3.8	[0.9]	3.8	[0.5]	4.4	[0.6]	4.1	[0.6]	4.3	[0.7]	4.1	[0.9]	4.4	[0.7]
Panel discussion	4.1	[0.7]	4.1	[0.7]	3.2	[1.0]	3.8	[1.1]	4.0	[0.8]	4.2	[0.5]	4.0	[0.5]	4.2	[0.7]	3.8	[1.0]	4.3	[0.6]
Innovation & cooperation processes	4.0	[0.7]	3.9	[0.7]	4.5	[0.7]	3.6	[0.8]	3.8	[0.5]	4.1	[0.7]	4.0	[0.5]	4.0	[0.8]	4.4	[0.5]	4.1	[0.8]
Success stories and lessons learned	4.4	[0.7]	4.3	[0.7]	4.2	[0.8]	4.3	[1.0]	4.0	[0.0]	4.4	[0.6]	4.0	[0.7]	4.4	[0.6]	4.2	[0.8]	4.8	[0.5]

### Benefits to participants

Overall, the top three benefits reported by participants were: i) sharing experiences and lessons learned (50 (75%)); ii) new opportunities for future collaboration (46 (69%)); and iii) new knowledge (45 (67%)). These three benefits were consistently the most frequently reported across all demographic categories (Table 4). Participants with the least experience with EIHP reported the most benefits, while the most experienced participants reported the least (by percentage). Most participants reported receiving multiple benefits from the meeting and no participant reported receiving no benefits, yet much fewer participants reported they intended to utilize the benefits in a meaningful way. Only 25% or less of meeting participants reported an intent to utilize any of the potential benefits highlighted in the questionnaire, with the exception of pursuing new opportunities for future collaboration (26 (39%)). Furthermore, the number of participants reporting a certain benefit did not directly correspond to participants’ intentions to utilize the benefit in their work in a meaningful way. For example, by comparison, few participants reported new skills as a benefit overall (31 (46%)), but 13 (19%) reported an intent to utilize new skills, the third highest percentage.

**Table 4:** Number and percentage of participants reporting benefits from attending the International Forum

**Benefits reported**																					**Intent to utilize benefits in a meaningful way**	
**What benefits did you gain from attending the International Forum? (select all that apply):**	**Total**		**First time at international meeting on evidence-informed health policymaking**		**From low- or middle-income country**		**Self-reported role**						**Years of experience with EIHP**						**Gender (Female)**		**Total**	
							**Policymaker**		**Journalist**		**Researcher**		**<1**		**1–9**		**10–19**					
Sharing experiences and lessons learned	50	(75%)	31	(78%)	35	(78%)	8	(73%)	3	(75%)	34	(85%)	8	(89%)	29	(67%)	7	(100%)	18	(78%)	9	(13%)
New opportunities for future collaboration, including professional development	46	(69%)	30	(75%)	34	(76%)	10	(91%)	3	(75%)	30	(75%)	9	(100%)	28	(65%)	4	(57%)	19	(83%)	26	(39%)
New knowledge	45	(67%)	28	(70%)	31	(69%)	7	(64%)	3	(75%)	32	(80%)	8	(89%)	25	(58%)	6	(86%)	17	(74%)	17	(25%)
Renewed motivation and sense of purpose	38	(57%)	23	(58%)	26	(58%)	5	(45%)	2	(50%)	28	(70%)	8	(89%)	23	(53%)	3	(43%)	14	(61%)	13	(19%)
Better understanding of the meaning and importance of evidence-informed policymaking	36	(54%)	23	(58%)	26	(58%)	8	(73%)	4	(100%)	20	(50%)	7	(78%)	18	(42%)	4	(57%)	13	(57%)	7	(10%)
Increased awareness of the challenges in evidence-informed policymaking	35	(52%)	21	(53%)	27	(60%)	6	(55%)	2	(50%)	23	(58%)	7	(78%)	20	(47%)	3	(43%)	13	(57%)	2	(3%)
Better understanding of how research can be utilized to inform health policy	31	(46%)	20	(50%)	25	(56%)	9	(82%)	2	(50%)	17	(43%)	6	(67%)	16	(37%)	4	(57%)	13	(57%)	10	(15%)
Affirmation of current work, approach and practice	27	(40%)	16	(40%)	19	(42%)	2	(18%)	2	(50%)	20	(50%)	5	(56%)	18	(42%)	1	(14%)	10	(43%)	5	(7%)
New skills	31	(46%)	19	(48%)	22	(49%)	5	(45%)	3	(75%)	22	(55%)	6	(67%)	21	(49%)	2	(29%)	12	(52%)	13	(19%)
Opportunity to advocate on specific issues	23	(34%)	17	(43%)	17	(38%)	6	(55%)	2	(50%)	14	(35%)	5	(56%)	13	(30%)	2	(29%)	10	(43%)	6	(9%)
Identification or clarification of priority needs and the ways I can help meet them	19	(28%)	10	(25%)	10	(22%)	6	(55%)	1	(25%)	11	(28%)	5	(56%)	7	(16%)	4	(57%)	7	(30%)	2	(3%)

### Qualitative comments on meeting organization

Twelve participants noted that the logistics and the pre-meeting organization could have been strengthened, specifically through reducing last minute changes to the programme and being clearer about meeting objectives. Multiple participants also reported that there was too much in the programme and that “less is more”, which would have provided more time for discussions. In terms of sessions participants enjoyed the most, seven participants highlighted presentations on country experiences and six participants highlighted the impact evaluation/analysis sessions as their favourites. Four participants highlighted a need to explore the sustainability of EIHP initiatives and how to finance policy research and initiatives. Finally, four other participants suggested expanding the capacity building sessions at the International Forum to include topics such as training on writing evidence briefs for policy.

## Discussion

This evaluation of the International Forum demonstrates that participants were highly satisfied with the various programme components. A majority of participants reported receiving multiple benefits but fewer participants reported an intent to utilize any of these benefits in their future work. It is interesting to note that there was no significant correlation between high ratings for any type of session (i.e., plenary, workshop, informal discussion) and benefits reported, suggesting that the meeting structure as a whole has a greater impact than the organization of one specific component. Overall, the evaluation findings suggest that the International Forum achieved its objectives of sharing experiences with EIHP and providing opportunities for networking among EIHP initiatives, though it is difficult to tell whether the meeting was successful in identifying opportunities to improve efforts to support EIHP in LMICs. There were some differences in findings between demographic categories, which were reported above, but generally, ratings were fairly consistent across categories. However, participants from LMICs did rate some of the plenary sessions lower and noted in their comments that more presentations from LMIC participants would have provided more relevant information. A similar concern was noted by policymakers and some comments suggested inviting more current policymakers.

Several strengths and weaknesses in this study should be considered. In terms of strengths, this is one of the first meeting evaluations to be developed based on a framework that incorporates theories and previous evaluative practices specific to large meetings. Further, our survey included both quantitative and qualitative components to increase the breadth of findings we could examine. We also achieved a fairly high response rate for an international meeting survey, at 55%. Finally, we conducted a formal analysis and synthesis of the data to add to a limited body of research on conference evaluations. A weakness of this study is that only one tool (a survey) was used for data collection, which could have been strengthened through intercept interviews to further explore some findings. Finally, though we achieved a high response rate for an international conference evaluation, the relatively small size of the meeting limited the total number of participants. As a result, performing reliable and generalizable regression analyses proved difficult.

This evaluation confirms that conferences and meetings can be successful in relation to traditional participant objectives such as sharing experiences, networking and gaining new knowledge but that there is still work to be done towards increasing meetings’ impact on its participants and associated sectors. In order to ensure that we are getting the most of out of these meetings, we need to continue to develop effective ways of evaluating them. Future evaluations of international meetings could build on the present study by expanding upon our methodology to include interviews or follow-up to supplement an end-of-conference survey. Furthermore, our previous scoping review on conference objectives and evaluations, as well as numerous other evaluation studies, highlight the importance of including not only participant perspectives but indicators of sector development in evaluations as well [4,6-8]. This study takes the first steps in this process by collecting information on a number of important EIHP activities, which can be compared with evaluation data at future iterations of the International Forum to track any progress in the field as a whole. This being said, there remain unanswered questions about the effectiveness of these indicators in predicting the impact of the meeting itself on the field of EIHP.

## Conclusions

The International Forum on Evidence Informed Health Policymaking successfully encouraged sharing experiences and lessons learned, and new opportunities for future collaboration and knowledge exchange between a variety of stakeholders. Furthermore, all participants said they would attend the International Forum again in the future. Meeting evaluations should continue to be made public to inform similar future meetings about successes and failures of the meeting itself, but also to strengthen and guide the development of evaluations themselves.

## Abbreviations

EIHP: Evidence Informed Health Policymaking; LMIC: Low- or middle-income country.

## Competing interests

UP is employed by the World Health Organization, a co-sponsor and organizer of the International Forum. MH was funded by the meeting organizers to administer the evaluation among other logistic duties. JN and JNL have no competing interests.

## Authors’ contributions

JN and JNL developed the framework and methodology for the evaluation. UP and MH organized the logistics and administration of the evaluation at the meeting. MH completed the initial compiling of the survey data. JN completed the formal data entry and analysis. JN also wrote the manuscript (including the figures and tables), with guidance from JNL. All authors reviewed the manuscript and provided their feedback. All authors read and approved the final manuscript.

## Supplementary Material

Additional file 1 **International Forum Questionnaire (English copy).** Description: A copy of the questionnaire handed out to meeting attendees. Click here for file
