# Supplementary material for: Evaluation of the international forum on evidence informed health policymaking: Addis Ababa, Ethiopia – 27 to 31 August 2012
Source: Health Res Policy Syst. 2014 Mar 19;12:14. doi: 10.1186/1478-4505-12-14 (PMC4004391; doi:10.1186/1478-4505-12-14)
Supplement: Additional file 1 — International Forum Questionnaire (English copy). Description: A copy of the questionnaire handed out to meeting attendees. [file 1478-4505-12-14-S1.doc]

**INTERNATIONAL FORUM ON EVIDENCE INFORMED HEALTH POLICYMAKING PARTICIPANT SURVEY**

**1. Programme: how useful were the following sessions for you?**

**1 = very poor; 2 = poor; 3 = satisfactory; 4 = good; 5= very good; N/A** = not applicable/did not attend

|  | **1** | **2** | **3** | **4** | **5** | **N/A** |
| --- | --- | --- | --- | --- | --- | --- |
| **Plenary sessions** |  |  |  |  |  |  |
| **Small group sessions** |  |  |  |  |  |  |
| **Poster presentations** |  |  |  |  |  |  |
| **Pre-forum workshop on Monday August 27^th^, 2012** |  |  |  |  |  |  |
| **Opening and closing dinners** |  |  |  |  |  |  |
| **Possibilities to have discussions and exchanges** |  |  |  |  |  |  |
| **Overall impression of the programme** |  |  |  |  |  |  |

Suggestions for the Programme

|  |
| --- |

**2. Evaluation of plenary sessions and small group sessions**

1 = very poor; 2 = poor; 3 = satisfactory; 4 = good; 5 = very good; N/A = not applicable/did not attend

|  | **1** | **2** | **3** | **4** | **5** | **N/A** |
| --- | --- | --- | --- | --- | --- | --- |
| **(Tuesday Morning, August 28^th^) Opening Plenary** |  |  |  |  |  |  |
| **(Tuesday Afternoon, August 28^th^) Plenary 2: Looking at EIHP Initiatives** |  |  |  |  |  |  |
| **(Wednesday Morning, August 29^th^) Plenary 3: Panel Discussion** |  |  |  |  |  |  |
| **(Thursday Morning, August 30^th^) Plenary 4: Innovations, Cooperation Processes and Country Team Sustainability** |  |  |  |  |  |  |
| **(Thursday Afternoon, August 30^th^): Closing Plenary: Success Stories and Lessons Learned** |  |  |  |  |  |  |

**Which small group sessions, in general, did you appreciate most**?

| **#1** |  |
| --- | --- |
| **#2** |  |

Which small group sessions, in general, did you like the least?

| **#1** |  |
| --- | --- |
| **#2** |  |

Why? Other comments?

|  |
| --- |

**3. What benefits did you gain from attending the International Forum? (Please select all that apply)**

- New knowledge
- New skills
- New opportunities for future collaboration, including professional development and career development
- Sharing experiences and lessons learnt
- Affirmation of current work, approach and practice
- Renewed motivation and sense of purpose
- Opportunity to advocate on specific issues
- Identification or clarification of priority needs and the ways I can help meet them
- Better understanding of the meaning and importance of evidence informed health policymaking
- Better understanding of how research can be utilized to inform health policy
- Increased awareness of the challenges in evidence informed health policymaking
- I did not gain anything from the conference
- Other: Please explain:

**Which , if any, of the above benefits will you apply in a meaningful way to your work after the Forum?**

| **#1** |  |
| --- | --- |
| **#2** |  |
| **#3** |  |

Details? Other comments?

|  |
| --- |

**4. Past experience in evidence informed health policymaking**

**If you are a policymaker, please refer to 4a.**

**If you are a researcher, please refer to 4b.**

**If you are a journalist, please refer to 4c.**

**4a. Policymakers**

**In the past three months, how many times was research evidence used in the health policy decisions in which you’ve been involved?**

| **0** | **1-4** | **5-9** | **10+** | **Not Applicable** |
| --- | --- | --- | --- | --- |

**In the past three months, how many times did you speak with researchers about the research evidence available to support policy decisions in which you’ve been involved?**

| **0** | **1-4** | **5-9** | **10+** | **Not Applicable** |
| --- | --- | --- | --- | --- |

**In the past three months, how many times did you speak with a journalist regarding the evidence supporting health policy decisions in which you’ve been involved?**

| **0** | **1-4** | **5-9** | **10+** | **Not Applicable** |
| --- | --- | --- | --- | --- |

**4b. Researchers**

**In the past three months, how many times did you undertake activities to inform health policy decisions?**

| **0** | **1-4** | **5-9** | **10+** | **Not Applicable** |
| --- | --- | --- | --- | --- |

**In the past three months, how many times did you speak with policymakers about the research evidence available to support the health policy decision in which they’re involved?**

| **0** | **1-4** | **5-9** | **10+** | **Not Applicable** |
| --- | --- | --- | --- | --- |

**In the past three months, how many times did you speak with journalists about the research evidence available to support their reporting about health issues?**

| **0** | **1-4** | **5-9** | **10+** | **Not Applicable** |
| --- | --- | --- | --- | --- |

**4c. Journalists**

**In the past three months, how many times have you searched for evidence to support your reporting on health issues?**

| **0** | **1-4** | **5-9** | **10+** | **Not Applicable** |
| --- | --- | --- | --- | --- |

**In the past three months, how many times did you ask policymakers about the research evidence available to support the health policy decisions in which they were involved?**

| **0** | **1-4** | **5-9** | **10+** | **Not Applicable** |
| --- | --- | --- | --- | --- |

**In the past three months, how many times did you speak with researchers about the research evidence available to support your reporting on health issues?**

| **0** | **1-4** | **5-9** | **10+** | **Not Applicable** |
| --- | --- | --- | --- | --- |

5. Future meetings

|  | **Yes** | **No** |
| --- | --- | --- |
| **If the International Forum on Evidence Informed Health Policymaking were to be held again in the future, would you attend?** |  |  |

Which topics do you think should be the focus of a future International Forums on Evidence-Informed Health Policymaking, if one were to be held?

| **#1** |  |
| --- | --- |
| **#2** |  |
| **#3** |  |

Which speaker would you like to see in the future, if another International Forum were to be held?

| **#1** |  |
| --- | --- |
| **#2** |  |
| **#3** |  |

6. Questions about you

| **How many days did you attend the Forum?** | **1** | **2** | **3** | **4** |
| --- | --- | --- | --- | --- |

|  | **Yes** | **No** |
| --- | --- | --- |
| **Is your main role a policymaker?** |  |  |
| **Is your main role a researcher?** |  |  |
| **Is your main role a journalist?** |  |  |
| **Is your main role a donor?** |  |  |
| **Are you from a low or middle-income country?** |  |  |
| **Were you funded by the Organizing Partners (WHO, SURE, IDRC etc) to attend this International Forum?** |  |  |
| **Is this your first time attending an international forum devoted to the topic of evidence-informed health policymaking?** |  |  |

| **Demographics** |  |  |  |  |
| --- | --- | --- | --- | --- |
| **For how many years have you worked in the field in supporting evidence informed health policymaking?** | **Less than 1** | **1-9** | **10-19** | **20+** |
| **What is your age?** | **16-26** | **27-40** | **41-50** | **50+** |
| **What is your gender?** | **Male** | **Female** | **Transgender** | |

**We greatly appreciate your support**

**Sincerely,**

**The Organizing Committee of the International Forum on Evidence-Informed Health Policymaking in Low and Middle-Income Countries**
